# Use of systems pharmacology modeling to elucidate the operating characteristics of SGLT1 and SGLT2 in renal glucose reabsorption in humans

**DOI:** 10.3389/fphar.2014.00274

**Published:** 2014-12-10

**Authors:** Yasong Lu, Steven C. Griffen, David W. Boulton, Tarek A. Leil

**Affiliations:** ^1^Quantitative Clinical Pharmacology, Clinical Pharmacology and Pharmacometrics, Exploratory Clinical and Translational Research, Bristol-Myers SquibbPrinceton, NJ, USA; ^2^Diabetes Development Center, Global Clinical Research, Bristol-Myers SquibbPrinceton, NJ, USA; ^3^Clinical Pharmacology and Pharmacometrics, Exploratory Clinical and Translational Research, Bristol-Myers SquibbPrinceton, NJ, USA

**Keywords:** systems pharmacology model, SGLT, dapagliflozin, renal glucose reabsorption, glucosuria, diabetes mellitus

## Abstract

In the kidney, glucose in glomerular filtrate is reabsorbed primarily by sodium-glucose cotransporters 1 (SGLT1) and 2 (SGLT2) along the proximal tubules. SGLT2 has been characterized as a high capacity, low affinity pathway responsible for reabsorption of the majority of filtered glucose in the early part of proximal tubules, and SGLT1 reabsorbs the residual glucose in the distal part. Inhibition of SGLT2 is a viable mechanism for removing glucose from the body and improving glycemic control in patients with diabetes. Despite demonstrating high levels (in excess of 80%) of inhibition of glucose transport by SGLT2 *in vitro*, potent SGLT2 inhibitors, e.g., dapagliflozin and canagliflozin, inhibit renal glucose reabsorption by only 30–50% in clinical studies. Hypotheses for this apparent paradox are mostly focused on the compensatory effect of SGLT1. The paradox has been explained and the role of SGLT1 demonstrated in the mouse, but direct data in humans are lacking. To further explore the roles of SGLT1/2 in renal glucose reabsorption in humans, we developed a systems pharmacology model with emphasis on SGLT1/2 mediated glucose reabsorption and the effects of SGLT2 inhibition. The model was calibrated using robust clinical data in the absence or presence of dapagliflozin (DeFronzo et al., 2013), and evaluated against clinical data from the literature (Mogensen, 1971; Wolf et al., 2009; Polidori et al., 2013). The model adequately described all four data sets. Simulations using the model clarified the operating characteristics of SGLT1/2 in humans in the healthy and diabetic state with or without SGLT2 inhibition. The modeling and simulations support our proposition that the apparent moderate, 30–50% inhibition of renal glucose reabsorption observed with potent SGLT2 inhibitors is a combined result of two physiological determinants: SGLT1 compensation and residual SGLT2 activity. This model will enable *in silico* inferences and predictions related to SGLT1/2 modulation.

## Introduction

In the kidney, plasma glucose is continuously filtered by glomeruli and reabsorbed along the proximal tubules. Under normal physiological conditions, the reabsorption is almost complete. The reabsorption is mediated primarily by two sodium-glucose cotransporters (SGLTs), SGLT1 and SGLT2. In the kidney, SGLT2 is located in the early part (S1/S2 segments) of the proximal tubules, and is recognized as a low affinity, high capacity pathway for renal glucose reabsorption. SGLT1, on the other hand, is located in the distal part (S3 segment) of the proximal tubules, and is characterized as a high affinity, low capacity pathway (Wright, 2001; Mather and Pollock, 2011). SGLT2 is believed responsible for 80–90% of renal glucose reabsorption, and SGLT1 for the rest (10–20%) in healthy humans under normal physiological conditions (DeFronzo et al., 2012). SGLT2 has been identified as a viable target for improving glycemic control in diabetes. Two potent and selective SGLT2 inhibitors, dapagliflozin and canagliflozin, have been approved by many regulatory agencies for treating type 2 diabetes mellitus (T2DM).

Given the overwhelming contribution (>80%) of SGLT2 to renal glucose reabsorption, it has been expected that SGLT2 inhibitors, at sufficient exposures, would reduce renal glucose reabsorption by over 80%. This expectation, however, appeared to be contradicted by the clinical observations that only 30–50% of inhibition in glucose reabsorption was achieved with dapagliflozin and canagliflozin (Komoroski et al., 2009a; Devineni et al., 2013; Washburn and Poucher, 2013). To explain this apparent contradiction, several hypotheses, from peculiar pharmacokinetics of an inhibitor in the kidney (Liu et al., 2012) to SGLT1 compensation (Haddish-Berhane et al., 2010; Maurer et al., 2011; Abdul-Ghani et al., 2013), have been proposed. These hypotheses are yet to be tested.

Recently, Hummel et al. (2011) used a quantitative *in vitro* electrophysiological study to generate hypotheses about the relative contributions of human SGLTs to renal glucose reabsorption. Hummel et al. found that the two human transporters have similar apparent affinity for D-glucose (5 mM for hSGLT2 vs. 2 mM for hSGLT1), and inferred that the capacity of hSGLT1 for renal glucose reabsorption may be over 50% of hSGLT2 under normal conditions in humans. As such, the difference in the contribution to renal glucose reabsorption between the two cotransporters may be less profound than previously perceived.

Despite a large body of research in SGLTs and renal glucose reabsorption, the quantitative understanding of the characteristics of these cotransporters in humans remains limited (Vallon, 2011). Assessments in this regard have largely relied on fragments of data, insufficient to account for all key variables (e.g., SGLTs activities, plasma glucose levels, pharmacokinetic profiles of SGLTs inhibitors), and empirical, static mathematical models that do not account for the dynamic processes of renal glucose filtration, reabsorption, and transfer along tubular lumen over time. Consequently, a quantitative, holistic characterization has not yet been formulated.

Systems pharmacology modeling is a powerful tool for data and knowledge integration and hypothesis testing, and for providing quantitative understanding of a pharmacological target or pathway and insights into “what-if” scenarios that may not be feasibly obtained experimentally. For SGLTs-mediated renal glucose reabsorption, Yamaguchi et al. reported simplified systems pharmacology models in mice (Yamaguchi et al., 2012) and rats (Yamaguchi et al., 2011), and Haddish-Berhane et al. (2010) presented a conference poster on a minimal systems pharmacology model in humans with limited evaluation against clinical data on dapagliflozin (Komoroski et al., 2009a).

This report presents a systems pharmacology model that was developed based on renal physiology and a robust clinical data set, with emphasis on SGLTs-mediated glucose reabsorption in the proximal tubules. The model was evaluated against several external clinical data sets. It is anticipated that the model will be valuable in:

1. Quantitatively evaluating the relative contributions of SGLT1 and SGLT2 to renal glucose reabsorption under various glucose load conditions in humans;
2. Explaining the apparently contradictory clinical observation that potent SGLT2 inhibitors only inhibit 30–50% of renal glucose reabsorption;
3. Mapping genetic mutations of renal SGLT2 to its *in vivo* activity and urinary glucose excretion (UGE); and
4. Predicting the effect of SGLT2 inhibition on glycemic control in diabetes mellitus where clinical data remain scarce, e.g., elderly and pediatric patients, and patients with type 1 diabetes mellitus (Lu et al., 2014).

## Materials and methods

### Studies and data sets

The studies and data sets used for model calibration and evaluation are listed in Table 1. For more details, the reader is referred to the original reports.

**Table 1:** **Studies and data sets used for model calibration and evaluation**.

**Study**	**Subjects**	**Study procedure**	**Data pertinent to modeling**	**Use**
DeFronzo et al., 2013	Healthy (*N* = 12), T2DM (*N* = 12)	SHC at baseline and after 7 daily doses of 10 mg dapagliflozin treatment; target plasma glucose level 100, 150, 200, 250, 300, 350, 400, 450, 500, and 550 mg/dL.	Dapagliflozin plasma concentration time course after the last dose; actual plasma glucose and iohexol concentrations, urine volume, urine glucose and iohexol concentrations at each step.	Model calibration
			Raw data available from BMS internal database.	
Polidori et al., 2013	T2DM (*N* = 28)	SHC at baseline and after 8 daily doses of 100 mg canagliflozin treatment; target blood glucose level 126, 171, 216, 261, and 306 mg/dL at baseline and 72, 117, 162, 207, and 252 mg/dL after treatment.	Canagliflozin plasma concentration time course in Devineni et al. (2013); Creatinine clearance, actual blood glucose, and UGE rate in Polidori et al. (2013).	Model evaluation
Mogensen, 1971	Healthy (*N* = 9), Diabetics (*N* = 10)	Plasma glucose escalated to over 650 mg/dL via glucose infusion.	GFR, plasma glucose concentration, and UGE rate in Mogensen (1971).	Model evaluation
Wolf et al., 2009	T2DM (*N* = 22)	SHC; target blood glucose level 140, 160, 180, 200, 220, 240 mg/dL.	GFR, actual blood glucose level, and tubular glucose reabsorption rate in Wolf et al. (2009).	Model evaluation

The DeFronzo et al. (2013), Polidori et al. (2013) and Wolf et al. (2009) studies employed stepped hyperglycemic clamp (SHC) procedures, and the Mogensen study (1971) was conducted at fixed, elevated plasma glucose levels. The clinical approach of artificially maintaining a constant plasma glucose concentration allowed us to ignore the potential impacts of renal glucose reabsorption on plasma glucose concentration, hence simplifying the process of model development. Simulations using the systems pharmacology model with fixed glucose levels will provide “clean” illustrations of SGLTs operating characteristics. A more comprehensive model integrating renal glucose reabsorption and glucose-insulin homeostasis will be reported elsewhere (Lu et al., 2014).

The mean data from each study were used for model calibration or evaluation. The data in DeFronzo et al. (2013) were available from an internal database owned by Bristol-Myers Squibb/AstraZeneca. We excluded from analysis those data points where the actual plasma glucose level deviated 25% or more from the corresponding group means. These data points appeared at the steps with target glucose level ≥450 mg/dL, and represented only 17% of total data points at those steps. This exclusion should abolish potential undue influences of excessive variability in the data on parameter estimation.

### Model structure

**Figure 1:**
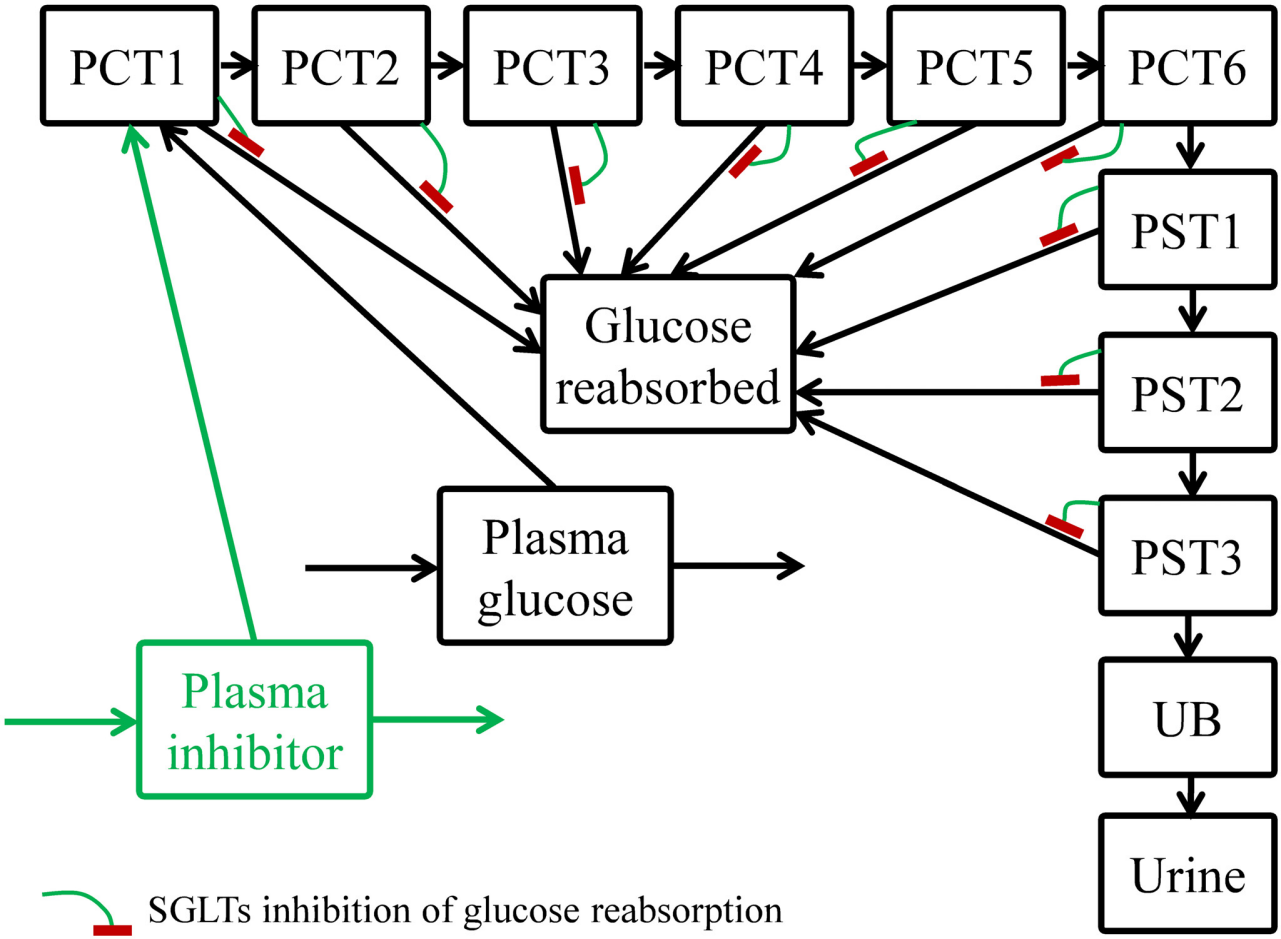
**Structure of the systems pharmacology model for describing renal glucose reabsorption and the inhibitory effect of an SGLTs inhibitor**. PCT1-6: sub-segments 1–6 of proximal convoluted tubules; PST1-3: sub-segments 1–3 of proximal straight tubules; UB, urinary bladder.

The model structure, shown in Figure 1, was developed based on the renal physiology and pharmacological understanding of SGLTs inhibition. The model describes the disposition of glucose as well as SGLTs inhibitors, if applicable, with emphasis on glomerular filtration and tubular reabsorption. The proximal convoluted tubules (PCT) were divided equally into six sequential sub-segments (PCT1-6), and the proximal straight tubules (PST) were divided equally into three sub-segments (PST1-3). The division allowed a more accurate description of the luminal glucose concentration as the filtrate progresses through tubular segments and the amount of UGE over time. The number of sub-segments was chosen to achieve an approximate agreement between predicted and observed UGE in a healthy subject under normal conditions. The distal tubules were not included due to their irrelevance to glucose reabsorption. The glomerular filtrate flowed from PCT1 through PST3 and drained into the urinary bladder. A urine compartment was added for collecting urine and urinary glucose.

Along the proximal tubules, filtered glucose was continuously reabsorbed. It was assumed that the absorption was mediated by SGLT2 in the PCT (PCT1-6) and by SGLT1 in the PST (PST1-3). The maximum reabsorption rate of SGLT2 (V_max_2) was uniformly distributed among the PCT1-6 sub-segments, and likewise for the V_max_ of SGLT1 (V_max_1) among the PST1-3 sub-segments. In each sub-segment, glucose was reabsorbed via a Michaelis-Menten process as Equation (1):

(1) $$R_{j}=\frac{V_{\max,j}\times C_{glu,j}}{K_{m}+C_{glu,j}}$$

where the subscript j is an index for a tubular sub-segment, and for a given sub-segment, *R* is the glucose reabsorption rate (mass/time), *K_m_* denotes glucose affinity for SGLT1 or SGLT2, and *C_glu_* represents luminal glucose concentration.

The mass of reabsorbed glucose was directed to another compartment (glucose reabsorbed) instead of the plasma glucose compartment. This approach is appropriate for scenarios where renal glucose recovery does not affect plasma glucose level, such as: (1) experimental procedures that fix plasma glucose levels (Mogensen, 1971; Wolf et al., 2009; DeFronzo et al., 2013; Polidori et al., 2013), and (2) subjects with normal glucose tolerance who can efficiently dispose the absorbed mass to maintain plasma glucose constant at the fasting state.

In the case where an SGLTs inhibitor, e.g., dapagliflozin or canagliflozin, was administered, the unbound portion of the inhibitor in plasma was freely filtered via glomeruli. The inhibitor then traveled through the tubular sub-segments and the urinary bladder, and was excreted to the urine compartment, similar to glucose but without tubular reabsorption. Within each sub-segment, the inhibitor competed with glucose for SGLT1/2, and hence competitively inhibited glucose reabsorption. The reabsorption rate (R^*^_j_), with competitive inhibition of an inhibitor, in a given sub-segment became:

(2) $$R_{j}^{*}=\frac{V_{\max,j}\times C_{glu,j}}{K_{m}\times \left(1+\frac{C_{drug,j}}{K_{i}}\right)+C_{glu,j}}$$

where *C_drug_* denotes luminal SGLTs inhibitor concentration, and *K_i_* is the affinity of the inhibitor to SGLTs. [See Supplementary Materials for further expansion of Equations (1) and (2)].

To obtain a similar time course of plasma concentration of canagliflozin with the dosing regimen (100 mg QD for 8 days) described in Polidori et al. (2013), a two-compartment pharmacokinetic (PK) model was developed based on the mean PK data reported (Devineni et al., 2013). For dapagliflozin, observed mean PK after 10 mg, QD, for 7 days was reported in DeFronzo et al. (2013). Interpolation of the observed dapagliflozin PK provided an input into the plasma inhibitor compartment (Figure 1) to allow description of dapagliflozin inhibition of tubular glucose reabsorption.

### Model parameters and calibration

The physiological parameters, such as volumes, flow rates, glucose affinity for SGLTs (K_m_), and glucose reabsorption capacities (V_max_) are listed in Table 2, and SGLT2 inhibitor physicochemical parameters and binding affinity for SGLTs (K_i_) are in Table 3. Most of the parameters were from the literature, measured in each of the respective studies, or based on reasonable assumptions, except for V_max_, K_m_, and K_i_, whose values were calibrated. For parameter calibration, literature values were taken as the starting points (see Tables 2, 3), and then fine-tuned to allow the model predictions to be consistent with the mean UGE data in DeFronzo et al. (2013). Because a satisfactory agreement between the predictions and the observations of UGE could not be achieved over the entire plasma glucose range of 100–550 mg/dL, the calibration was focused on the data in the clinically relevant range, 100–400 mg/dL. In the end, only the set of calibrated values were considered physiologically plausible and accepted if it adequately described the DeFronzo et al. (2013) as well as the other three data sets (Mogensen, 1971; Wolf et al., 2009; Polidori et al., 2013).

**Table 2:** **Physiological parameters**.

**Parameter**	**Symbol (unit)**	**Value**	**Rationale and/or Reference**
**VOLUMES**			
Renal cortex volume	VCTX (L)	0.216	Thelwall et al., 2011
Proximal tubules (PT) volume as a fraction of renal cortex	VPTC	0.3	Moller and Skriver, 1985
PCT volume as a fraction of PT	VPCTC	0.7	Assumed
PST volume as a fraction of PT	VPSTC	1–VPCTC	
Urinary bladder volume	VX (L)	0.2	Brown et al., 2011
**FLOW RATES**			
Glomerular filtration rate (GFR)	GFR (L/h)		
DeFronzo et al., healthy baseline		5.66–7.38	Measured using iohexol as a marker, raw data from an internal database owned by Bristol-Myers Squibb/AstraZeneca
DeFronzo et al., healthy after dapagliflozin		5.19–7.41	
DeFronzo et al., T2DM baseline		6.52–7.62	
DeFronzo et al., T2DM after dapagliflozin		5.07–7.53	
Healthy and diabetic subjects in other studies		Various	Mogensen, 1971; Wolf et al., 2009; Polidori et al., 2013
Filtrate flow rate in tubular lumen	KPCi (L/h) for PCT, where *i* = 1–6; KPSj (L/h) for PST, where *j* = 1–3	From 0.926 × GFR to 0.333 × GFR with decrements of 0.074 × GFR for PCT1 to PST3	Calculated based on (1) 2/3 of filtered water is reabsorbed by the end of PT (Koeppen and Stanton, 2013), and (2) the assumption that the water reabsorption rate is identical in all proximal tubular sub-segments.
Rate of flow out of urinary bladder	KX (L/h)		
DeFronzo et al., healthy baseline		0.63–1.20	Measured, raw data from an internal database owned by Bristol-Myers Squibb/AstraZeneca
DeFronzo et al., healthy after dapagliflozin		0.78–1.40	
DeFronzo et al., T2DM baseline		0.54–1.24	
DeFronzo et al., T2DM after dapagliflozin		0.80–1.20	
Polidori et al., T2DM, Mogensen healthy and diabetics		0.60	Assumed based on observations in the DeFronzo et al., 2013
Wolf et al., diabetics		0.28	Wolf et al., 2009
**GLUCOSE REABSORPTION**			
SGLT1 maximum reabsorption rate	V_max_1 (mmol/h)	20.0	Model calibration (10% of 140 mmol/h in T2DM patients DeFronzo et al., 2013 as starting point)
SGLT2 maximum reabsorption rate in diabetics	V_max_2 (mmol/h)	110.0	Model calibration (90% of 140 mmol/h in T2DM patients DeFronzo et al., 2013 as starting point)
SGLT2 maximum reabsorption rate in healthy	V_max_2 (mmol/h)	93.5	Model calibration (100% of Vmax2 in diabetes as starting point)
Glucose affinity for SGLT1	K_m_1 (mM)	0.5	Model calibration (1.8 mM from Hummel et al., 2011 as starting point)
Glucose affinity for SGLT2	K_m_2 (mM)	4.0	Model calibration (4.9 mM from Hummel et al., 2011 as starting point)

**Table 3:** **SGLT2 inhibitor-specific parameters**.

**Parameter**	**Dapagliflozin**	**Canagliflozin**
Molecular weight (MW, g/mole)	409	454
Free fraction in plasma (fup)	0.07^*^	0.01 Devineni et al., 2013
Affinity for SGLT1 (K_i_1, nM)	400 Hummel et al., 2011	200 (half of dapagliflozin Ki1 as per Grempler et al., 2012)
Affinity for SGLT2 (K_i_2, nM)	0.3 (model calibration, 6 nM from Hummel et al., 2011 as starting point)	0.6 (2-fold of dapagliflozin Ki2 as per Grempler et al., 2012)

* *Reference: Bristol-Myers Squibb/AstraZeneca report (2010): Summary of clinical pharmacology studies: Dapagliflozin (BMS-512148). BMS Document Control Number 930047848*.

The potential influences of diabetes and SGLT2 inhibition on the parameters to be calibrated were considered during parameterization. Renal SGLTs expression and activity may change in response to SGLT2 inhibition and/or diabetes. In the wild-type mouse, SGLT2 protein expression was enhanced with the treatment of empagliflozin, a selective SGLT2 inhibitor, without upregulation of mRNA (Vallon et al., 2014). In the diabetic state, the expressions of SGLT2 mRNA and protein have been found upregulated significantly relative to the respective controls in genetically modified mice (Vallon et al., 2014), diabetic rats, (Freitas et al., 2008; Tabatabai et al., 2009), and humans (Rahmoune et al., 2005). For renal SGLT1, however, the response is more diverse, with increased, unchanged, or reduced expression and/or activity observed in animals (Vallon and Thomson, 2012; Vallon et al., 2014). It is challenging to incorporate these potential changes in SGLT activity in the model for two reasons: (i) limited quantitative understanding in humans regarding these changes, and (ii) adequate calibration of parameters for these changes is not supported by available data. For simplification, therefore, the V_max_1, K_m_, and K_i_ values were assumed consistent between the healthy and diabetics, and V_max_2 was allowed to adjust between the healthy and disease state. The V_max_2 in healthy subjects was estimated as a proportion of that in diabetics, and the value of the proportion was calibrated using the DeFronzo et al. (2013). The potential impact of SGLT2 inhibition on V_max_, K_m_, and K_i_ values was ignored.

Although V_max_ in humans has generally been reported as the sum of V_max_1 and V_max_2, with difficulty in separating the two components, it is worth pointing out that in our study, quantitative separation of V_max_1 and V_max_2 was feasible without an assumption of the value of V_max_1/V_max_2 ratio, because the calibration data set (DeFronzo et al., 2013) encompassed scenarios with and without perturbation of SGLT2 activity. Such a separation was achieved previously in rats with the aid of mathematical modeling (Yamaguchi et al., 2011).

### Model evaluation

Once it was calibrated using the DeFronzo et al. (2013), the model was evaluated for its predictivity against three data sets from different sources (Mogensen, 1971; Wolf et al., 2009; Polidori et al., 2013). The parameters were held constant for the evaluation unless they were study specific, in which case they were adjusted per the study conditions as listed in Tables 1, 2. The K_i_ values of canagliflozin, necessary for simulating the Polidori et al., conditions (Polidori et al., 2013), are listed in Table 3.

### Simulations and explorations

#### Renal glucose reabsorption and UGE vs. loss-of-function mutation of SGLTs

Numerous mutations in SGLT1 (Martin et al., 1996; Lam et al., 1999) and SGLT2 have been identified in humans (Santer et al., 2003; Kleta et al., 2004; Calado et al., 2008; Yu et al., 2011). The mutations in SGLT1 disrupt the trafficking of SGLT1 from the endoplasmic reticulum to the plasma membrane (Lam et al., 1999), and the mutations in SGLT2 reduce SGLT2 expression in the apical side of PCT (Yu et al., 2011). These mutations are likely to reduce the V_max_ of these cotransporters. It is yet to be clarified to what extent the function of SGLTs in the kidney is affected by a given mutation. Simulations using our systems pharmacology model can provide theoretical, quantitative relationships between a reduction in V_max_ and glucose reabsorption or UGE in an otherwise healthy person. To enable these simulations, the mean daily plasma glucose profile in the healthy subjects from Freckmann et al. (2007) was used as an input to the plasma glucose compartment of our model. The quantitative SGLTs function-UGE relationships will be instrumental to mapping renal SGLTs genotypes to their apparent functions.

#### Sensitivity of renal glucose reabsorption and UGE to SGLT1 kinetics (V_max_1 and K_i_1)

The analysis of sensitivity of renal glucose reabsorption and UGE to SGLT1 kinetics will help clarify these questions: (1) How strong is the influence of an alteration of SGLT1 kinetics on renal glucose reabsorption and UGE in the healthy state? (2) How strong is the influence in the diabetic state? (3) From drug discovery perspective, without consideration of its effect on intestinal SGLT1, will an SGLT1/2 dual inhibitor induce stronger glucosuria than a highly selective SGLT2 inhibitor, e.g., dapagliflozin? The analysis was conducted with simulations in a naive healthy subject and a T2DM subject with or without SGLT2 inhibition under the SHC procedure used by DeFronzo et al. (2013) with a target plasma glucose range of 100–350 mg/dL. With all other parameters held constant, we first evaluated how a decrease in V_max_1 would affect renal glucose reabsorption and UGE; and likewise, we then evaluated how changes in K_m_1, or K_i_1 with the presence of SGLTs inhibition, would affect renal glucose reabsorption and UGE.

### Software

Processing of the raw data from the DeFronzo et al. study (2013) was conducted using S-PLUS 8.1 version 3.4 (TIBCO, Palo Alto, CA) on a UNIX platform. Model development and simulations were performed using Berkeley Madonna version 8.3.18 (Berkeley Madonna Inc., Berkeley, CA).

## Results

### Model calibration using DeFronzo et al. (2013)

**Figure 2:**
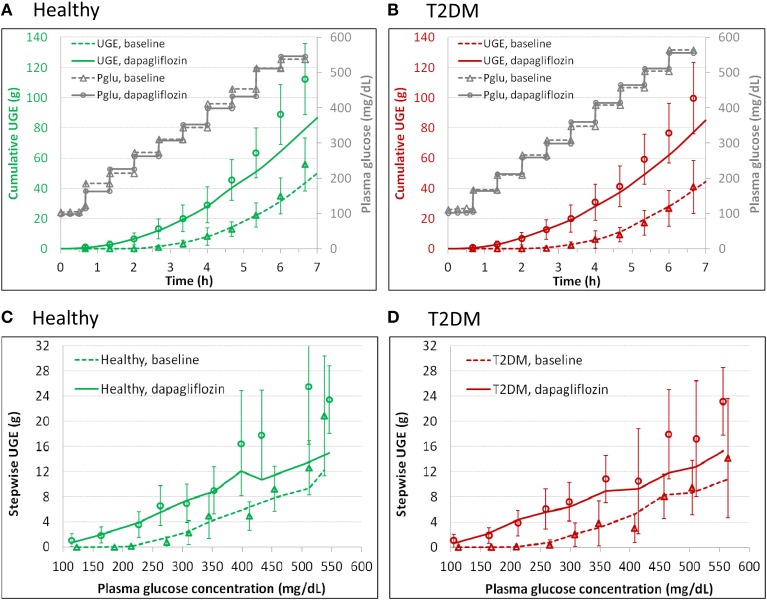
**Model description of cumulative (A,B) and step-wise (C,D) urinary glucose excretion (UGE) in the healthy (A,C) and T2DM (B,D) subjects at baseline and after 7 daily doses of 10 mg dapagliflozin in the DeFronzo et al. study (2013), where an stepwise hyperglycemic clamp procedure was employed**. The symbols represent observations and the curves are model predictions. Pglu, plasma glucose concentration.

The UGE data from DeFronzo et al. (2013) allowed estimation of V_max_, glucose K_m_, and dapagliflozin K_i_ values for SGLTs in the healthy subjects and T2DM patients. In the model, the healthy and T2DM subjects were differentiated by their V_max_2 for describing the DeFronzo et al. conditions. The parameter estimates are presented in Tables 2, 3. The model performance is demonstrated in Figure 2. The model adequately described the cumulative (Figures 2A,B) and step-wise UGE data (Figures 2C,D) at baseline and in the first 4 h (where the target plasma glucose escalated from 100 to 350 mg/dL) after dapagliflozin treatment. From 4.67 h onward (where the target plasma glucose increased from 400 to 550 mg/dL), the model prediction of UGE in the dapagliflozin treated groups was slightly lower than the observed. The glucose concentrations in the tubular sub-segments PCT1-6 and PST1-3 in T2DM patients at baseline and treated with dapagliflozin are illustrated in Figure S1. At baseline, the tubular glucose concentration tapers along the proximal tubules with plasma glucose level up to 23 mM. With further increase in plasma glucose, the tubular glucose level becomes more uniform as the reabsorption approaches saturation. After dapagliflozin treatment, however, glucose is increasingly concentrated along the proximal tubules.

### Model evaluation

**Figure 3:**
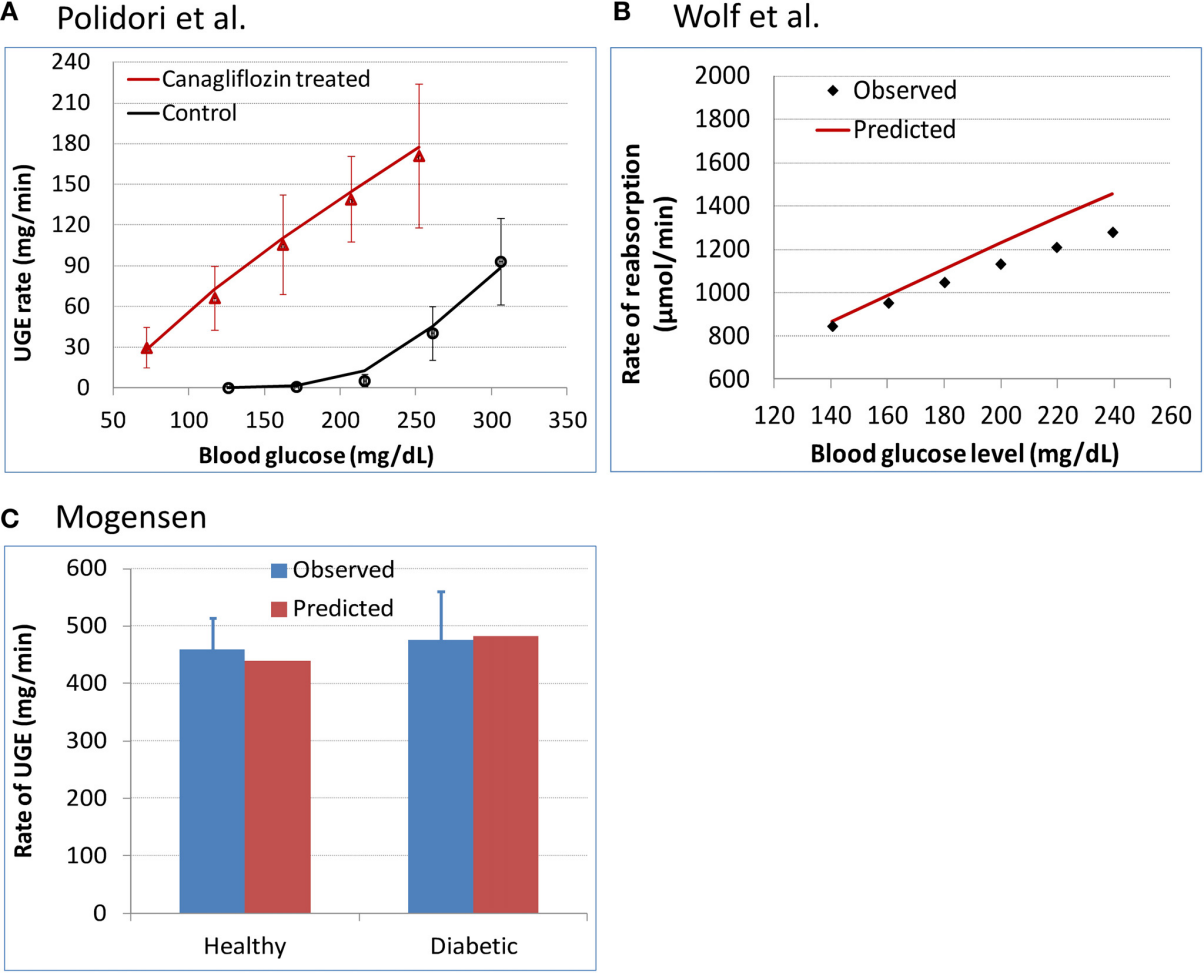
**Evaluation of model predictivity against three separate clinical data sets. (A)** Polidori et al. (2013) urinary glucose excretion (UGE) data in T2DM subjects at baseline and after 8 daily doses of 100 mg canagliflozin. The symbols represent the observed data and the curves are model predictions. **(B)** Wolf et al. (2009) renal glucose reabsorption rate in T2DM patients who were subjected to a stepwise hyperglycemic clamp procedure. **(C)** Mogensen (1971) renal glucose reabsorption rate in healthy and diabetic subjects with plasma glucose levels elevated to over 650 mg/dL.

The calibrated model was evaluated for its predictive performance relative to three separate clinical data sets (Mogensen, 1971; Wolf et al., 2009; Polidori et al., 2013). The predictions are overlaid with corresponding observations in Figure 3. The predictions agreed well with the observed data, indicating that the model is plausible, has reasonable accuracy, and can be used for inference and prediction.

### SGLTs operating characteristics for the DeFronzo et al. (2013) conditions

#### SGLTs relative contributions to renal glucose reabsorption

**Figure 4:**
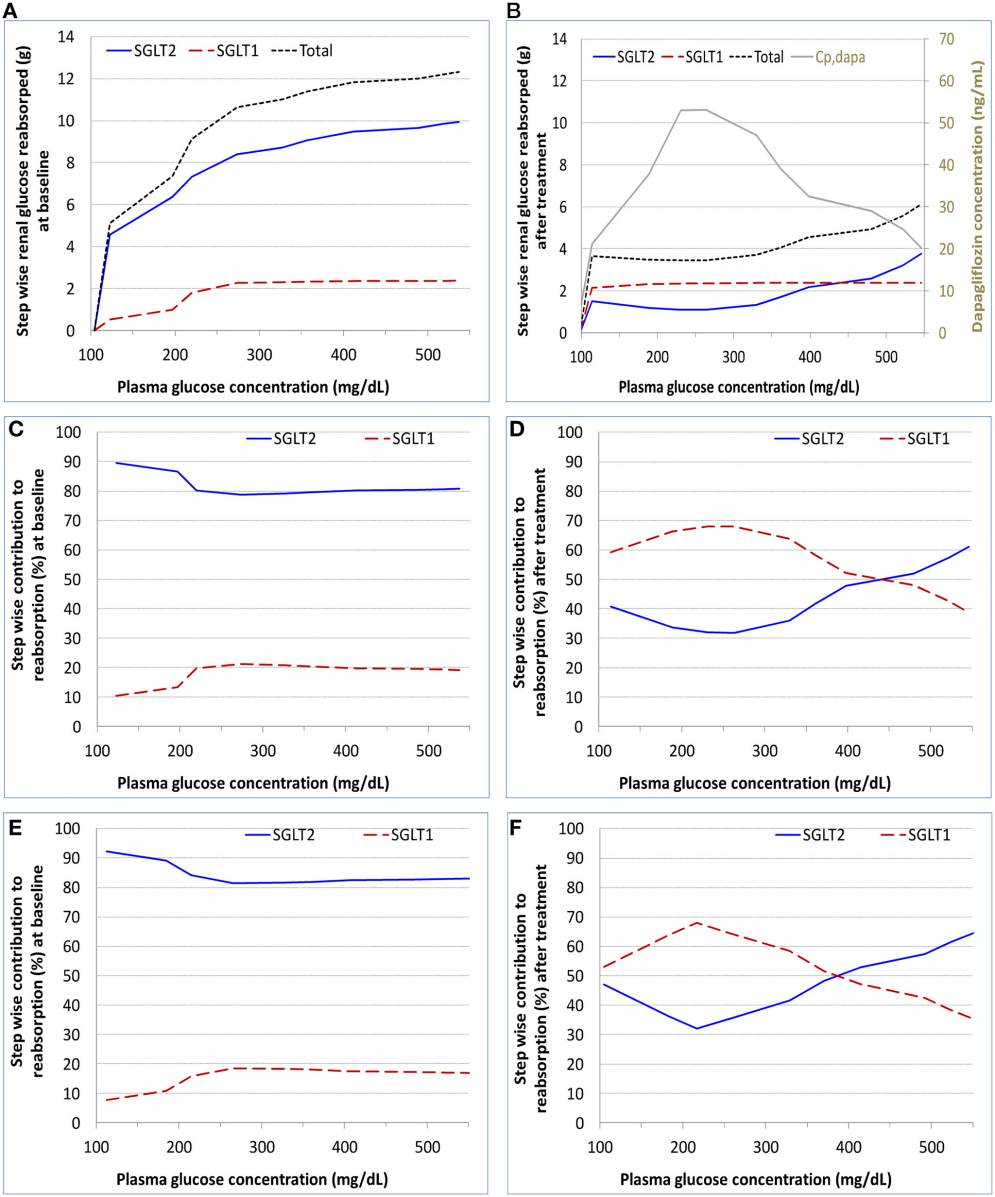
**Model calculated step-wise amount of glucose reabsorbed (A,B) and relative contributions to the reabsorption (C–F) by renal SGLT1 and SGLT2 at baseline and after dapagliflozin treatment in healthy subjects and patients with diabetes under the SHC procedure in DeFronzo et al. (2013). (A,C)** healthy, baseline; **(B,D)** healthy, after treatment; **(E)** patient, baseline; and **(F)** patient, after treatment. Cp,dapa, total plasma concentration of dapagliflozin.

The model derived step-wise amount of glucose reabsorbed by renal SGLT1 and SGLT2 in the healthy subjects at baseline and after dapagliflozin treatment is shown in Figures 4A,B, and the relative contributions of the two pathways at each step are in Figures 4C,D. At near normal glycemic levels (~100 mg/dL) at baseline (without SGLT2 inhibition), SGLT2 contributed to 90% of total reabsorption and SGLT1 10%. The 90%/10% split became 80%/20% with plasma glucose escalated to over 200 mg/dL. With the presence of dapagliflozin, the contribution of SGLT2 declined and SGLT1 became the more predominant reabsorption pathway; the relative contributions varied with plasma dapagliflozin concentration over time. Similar results were obtained in the T2DM patients, for whom the relative contributions of SGLT1 and SGLT2 before and after dapagliflozin treatment are illustrated in Figures 4E,F.

#### SGLTs operation efficiency

**Figure 5:**
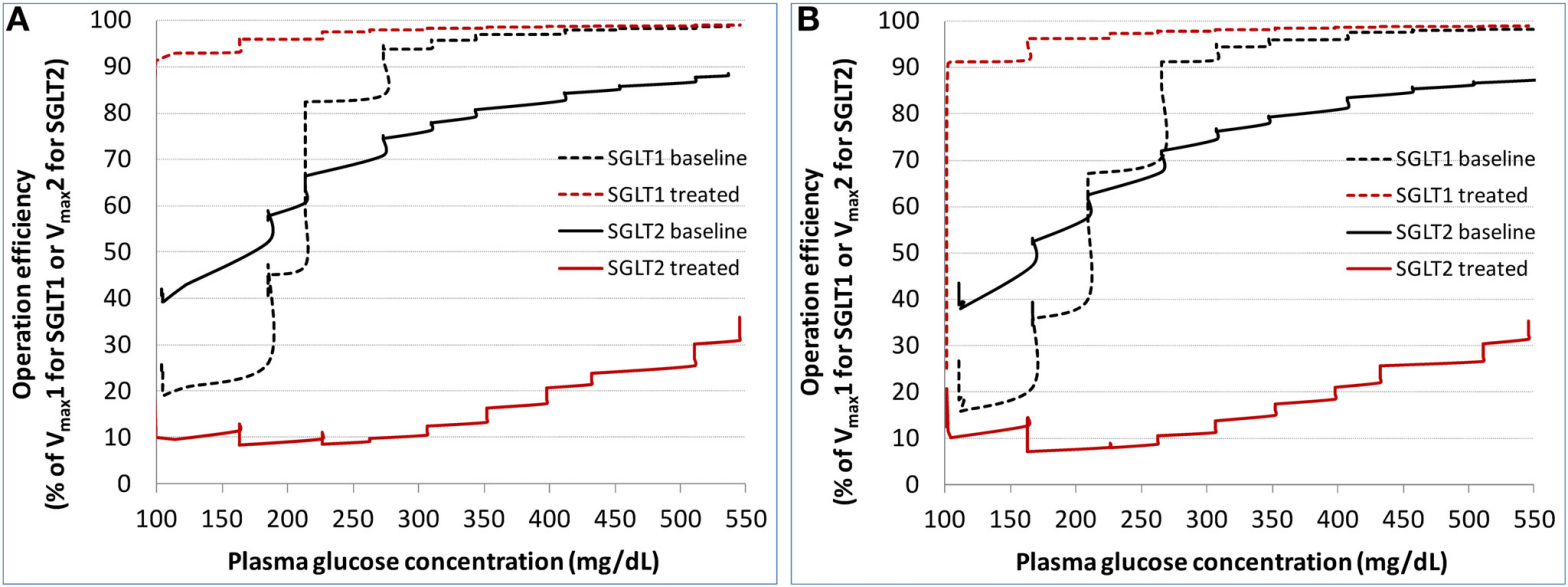
**Model derived operation efficiency (defined as glucose reabsorption rate/V_max_ × 100% for either SGLT1 or SGLT2) for both SGLTs at baseline and after dapagliflozin treatment in the healthy subjects (A) and patients with diabetes (B) under the stepwise hyperglycemic clamp procedure in DeFronzo et al. (2013)**.

The calculated operation efficiency (defined as reabsorption rate/V_max_ × 100% for either SGLT1 or SGLT2) for both SGLTs in the healthy subjects is plotted in Figure 5A. At the plasma glucose level of ~100 mg/dL, SGLT2 and SGLT1 were operating at ~40 and 20% of their respective V_max_. The operation efficiency increased with plasma glucose (and thereby filtered glucose load) for both SGLTs, with the slope for SGLT1 being much steeper than for SGLT2. The operation efficiency at plasma glucose ≥400 mg/dL reached 97% for SGLT1 and 81% for SGLT2. Even with plasma glucose as high as 550 mg/dL, SGLT2 operated at just 89% of its capacity. Dapagliflozin treatment lowered SGLT2 operation efficacy to as low as 10%, and drove SGLT1 operation to over 90% of its capacity. Similar results were found in the T2DM patients (Figure 5B).

### Simulations and explorations

#### Renal glucose reabsorption and UGE vs. loss-of-function mutation of SGLTs

**Figure 6:**
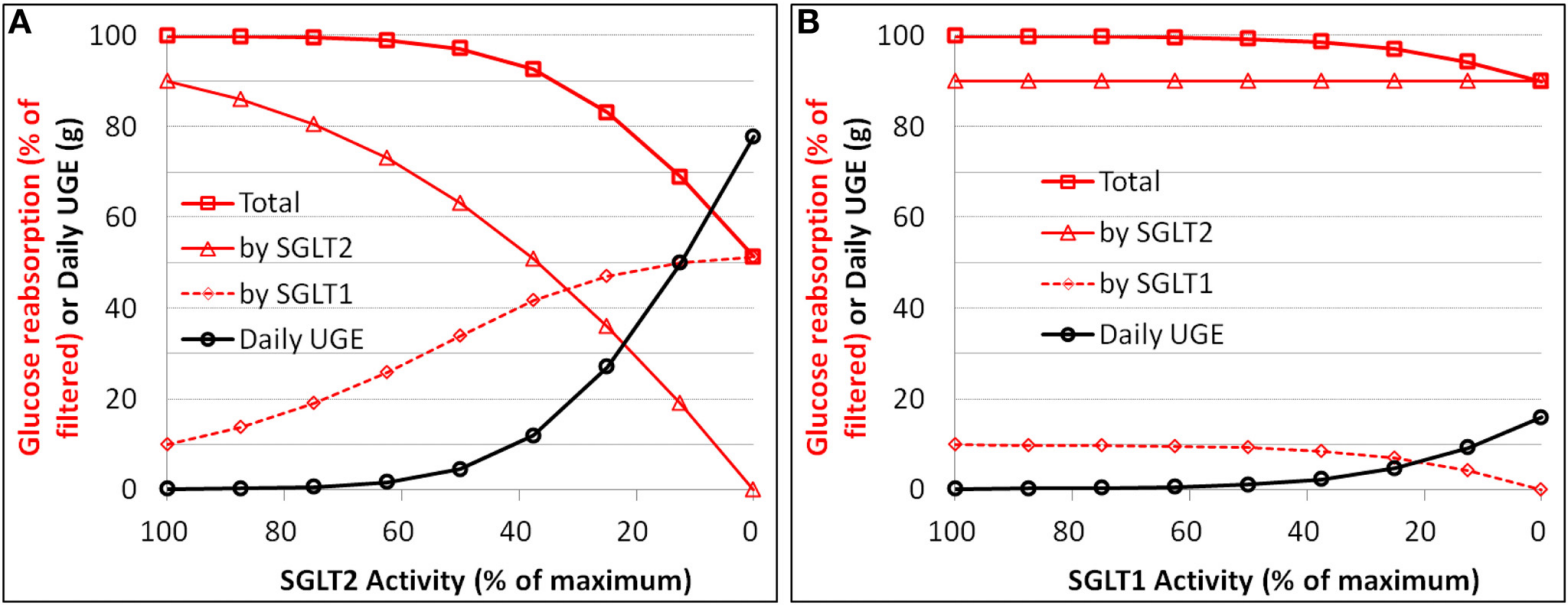
**Simulation of the relationships between loss of function (i.e., reduction in V_max_) and renal glucose reabsorption (% filtered) as well as urinary glucose excretion (UGE) for SGLT2 (A) and SGLT1 (B) in an otherwise healthy subject with normoglycemia**.

Simulations were conducted to establish quantitative relationships between loss of function (i.e., reduction in V_max_) of SGLT2 or SGLT1 and renal glucose reabsorption as well as UGE in an otherwise healthy subject with normoglycemia (plasma glucose ranging from 80 to 125 mg/dL with a time-weighted average of 90 mg/dL). The simulation results for SGLT2 are in Figure 6A and SGLT1 in Figure 6B. A 50% loss of function for SGLT2 caused UGE of 4.5 g per day, and 100% of loss of function resulted in 79 g UGE per day. The total glucose reabsorption was lowered by 17, 32, and 49% for a 75, 87.5, and 100% loss of SGLT2 function, respectively. Loss of SGLT1 function caused much less severe glucosuria, 1.2 g at 50% and 16 g at 100% of loss of function. The total glucose reabsorption was reduced by only 10% with complete loss of SGLT1 activity.

#### Sensitivity of renal glucose reabsorption and UGE to SGLT1 kinetics

In a naive healthy subject with a plasma glucose level of ~100 mg/dL, the elimination of SGLT1 activity, either through driving V_max_1 to zero or K_m_1 to infinity, inhibited renal glucose reabsorption by only 10%. This was consistent with the result above for SGLT1 loss-of-function mutation (Figure 6B). In the diabetic state with a mean plasma glucose level up to 250 mg/dL, simulations suggested a slightly stronger influence (up to 20% lowering) on renal glucose reabsorption.

**Figure 7:**
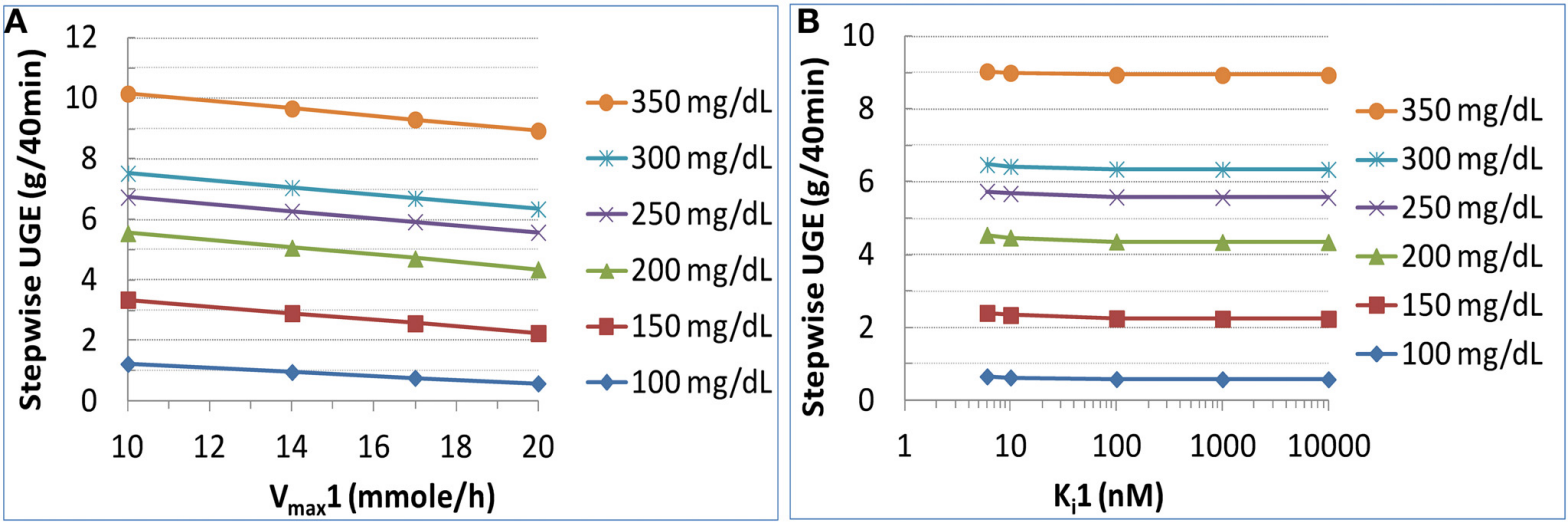
**Sensitivity of urinary glucose excretion (UGE) to SGLT1 capacity (V_max_1) (A) and inhibitor affinity to SGLT1 (K_i_1) (B) in a T2DM patient subjected to the same study procedure as in DeFronzo et al. (2013) with target plasma glucose from 100 to 350 mg/dL with increments of 50 mg/dL**. For the analysis on V_max_1, all other parameters were held constant and V_max_1 was varied to 10, 14, 17, or 20 mmole/h (corresponding to a 50, 30, 15%, or 0% reduction of SGLT1 capacity). For the analysis on K_i_1, the K_i_1 value of an SGLT2 inhibitor which was otherwise identical to dapagliflozin was varied from 6 to 10,000 nM, representing a selectivity for SGLT2 from 20× to 33,333×.

For simulations in the diabetic state with the presence of SGLT2 inhibition, the inhibitor was assumed identical to dapagliflozin, except that its K_i_1 was allowed to change. The sensitivity of UGE to V_max_1 at 10, 14, 17, and 20 mmol/h (corresponding to a 50, 30, 15, and 0% reduction in SGLT1 capacity) is illustrated in Figure 7A. A mild to moderate, depending on the glucose level, increase in UGE was expected with reduction in V_max_1. At the plasma glucose level of 150–250 mg/dL, roughly equivalent to the range of average levels in real-life T2DM patients, a 30% reduction in V_max_1, presumed to be clinically well tolerated (Abdul-Ghani et al., 2013), augmented glucosuria by up to 30%. The sensitivity of UGE to K_i_1 is depicted in Figure 7B. The tested K_i_1 values ranged from 6 to 10,000 nM. The 6 nM represented a 20× selectivity (similar to the SGLT1/2 dual inhibitor LX4211 Zambrowicz et al., 2013) for SGLT2 (0.3 nM) vs. SGLT1 (6 nM) for an SGLTs inhibitor which is otherwise identical to dapagliflozin. UGE was found to be insensitive to K_i_1.

## Discussion

Highly selective and potent SGLT2 inhibitors, such as dapagliflozin and canagliflozin, have demonstrated significant and clinically meaningful effects on glycemic control in T2DM patients. It has been puzzling that SGLT2 inhibitors inhibit renal glucose reabsorption by only 30–50% clinically, despite the overwhelming contribution of SGLT2 to renal glucose reabsorption (80–90%) under normal conditions. Several hypotheses have been proposed for this apparently discrepant observation (Haddish-Berhane et al., 2010; Maurer et al., 2011; Pfister et al., 2011; Liu et al., 2012; Abdul-Ghani et al., 2013), and most of them are focused on the compensatory effect of SGLT1. The hypothesis of SGLT1 compensation has recently been confirmed in mice (Rieg et al., 2014). However, due to the differences in the experimental conditions in mice (Rieg et al., 2014) and in clinical trials (Komoroski et al., 2009a; Devineni et al., 2013; Heise et al., 2013; Washburn and Poucher, 2013) (see Table S1), extrapolation of the Rieg et al. (2014) finding to the clinic is not straightforward. As a whole, this situation indicates that, despite tremendous advances in the basic biology of SGLTs and pharmaceutical development targeting SGLT2, the roles of these transporters in renal glucose reabsorption, especially in humans, have yet to be clarified in a quantitative, mechanistic manner. To this end, we developed a systems pharmacology model for SGLT-mediated renal glucose reabsorption in humans with or without pharmacological modulation of SGLT2 activity. In general, this model adequately described four separate data sets from different study settings (Mogensen, 1971; Wolf et al., 2009; DeFronzo et al., 2013; Polidori et al., 2013) and replicated severe glucosuria (79 g/day) in normoglycemic human subjects with homozygous SLGT2 mutations (Santer et al., 2003).

The prediction of UGE at the plasma glucose level of 400 mg/dL and higher in the subjects treated with dapagliflozin was lower than the observed data from DeFronzo et al., (Figure 2). This possibly results from compensatory effects in the renal tubules when glucose concentrations are drastically elevated. Bank and Aynedjian (1990) proposed that high glucose concentration in the proximal tubules would stimulate water reabsorption in the proximal portion and enhance compensatory water excretion in the more distal portion. In the DeFronzo et al. study (2013), an increase in urine volume was observed with escalation of plasma glucose level. This hydrodynamic change in response to glucose level may interfere with tubular glucose reabsorption. These processes, however, were not included in the model. Nevertheless, the unsatisfactory performance at high glucose levels (over 400 mg/dL) is unlikely to hamper the utility of the model because those glucose levels are irrelevant to most of normal or even diabetic conditions. Overall, the performance of the model suggests that the model is useful for mechanistically evaluating the roles of SGLT1 and SGLT2 in renal glucose reabsorption, and for predicting clinical pharmacodynamics of SGLT2 inhibitors.

### Characterization of SGLTs operation without the presence of SGLT2 inhibition

The V_max_ values of SGLT1 and SGLT2 were estimated to be 20 mmol/h and 94 (healthy)/110 (diabetic) mmol/h, respectively, and the glucose K_m_ values for SGLT1 and SGLT2 were estimated to be 0.5 and 4 mM, respectively. The sum of the V_max_ values (i.e., total reabsorption capacity) and the two K_m_ estimates are similar to previously reported estimates (Mogensen, 1971; Diez-Sampedro et al., 2001; Chao and Henry, 2010; Hummel et al., 2011; DeFronzo et al., 2013). The V_max_2/V_max_1 ratio in the healthy subject (4.7) is consistent with that in the rat (5.4) (Yamaguchi et al., 2011). These estimates reinforce the concept of SGLT1 being a high affinity, low capacity transporter and SGLT2 being a low affinity, high capacity transporter for renal glucose reabsorption.

Under near normoglycemic conditions (average plasma glucose ~80–120 mg/dL) in both healthy and diabetic subjects, SGLT2 and SGLT1 are operating at about 40 and 20% of their respective capacities, and contributing to 90 and 10% of total glucose reabsorption, respectively. With the increase in plasma glucose concentration, SGLT2 operation efficiency steadily increases to near 90% of its capacity, whereas SGLT1 operation efficiency jumps sharply to over 80% of its capacity and then steadily approaches to the maximum. The relative contributions of 90%/10% gradually becomes 80%/20% for SGLT2 and SGLT1 as plasma glucose rises. These results solidify the current characterization of the relative contributions of the two transporters to renal glucose reabsorptoin without the presence of SGLT2 inhibition (Chao and Henry, 2010; DeFronzo et al., 2012).

### Characterization of SGLT's operation in the presence of SGLT2 inhibition

With the treatment of dapagliflozin at its clinical dose (10 mg QD), the majority of SGLT2 is occupied by dapagliflozin molecules (occupancy up to 98% at the peak exposure). The total activity of SGLT2 in a healthy or T2DM subject is suppressed considerably, from ~40% of operation efficiency without SGLT2 inhibition to only 10% with the treatment of dapagliflozin. Consequently, the contribution of SGLT2 to renal glucose reabsorption declines from 80 to 90% at baseline to less than 50% with SGLT2 inhibition. Meanwhile, the importance of SGLT1 to renal glucose reabsorption jumps sharply. The operation efficiency of SGLT1 reaches over 90%, up from 20% at baseline. As a result, SGLT1 accounts for over 50% of renal reabsorption when SGLT2 is inhibited, much higher than the 10–20% at baseline.

### Theoretical maximum inhibition of renal glucose reabsorption

The simulations of loss of function of SGLTs (reduction in V_max_) vs. renal glucose reabsorption provide a clean relationship for assessing the theoretical maximum inhibition of the reabsorption. In a healthy subject under physiological conditions, an 87.5–100% loss of SGLT2 function results in a 32–49% of inhibition of renal glucose reabsorption. In a diabetic patient, the glucose reabsorption vs. loss of SGLT2 activity curve shifts downwards, i.e., somewhat greater inhibition of reabsorption. With a daily average plasma glucose level of 150 mg/dL, a complete loss of SGLT2 activity lowers the reabsorption by 70%. This greater extent of inhibition in diabetics is due to the up-regulated activity of SGLT2 in the disease state (Rahmoune et al., 2005).

The loss of SGLT1 function has only mild inhibitory effect on renal glucose reabsorption. An entire loss of SGLT1 function leads to only 10% of inhibition of glucose reabsorption in a normoglycemic healthy subject and up to 15% of inhibition in a diabetic patient with a daily average plasma glucose level of 150 mg/dL.

Our results of theoretical maximum inhibition of renal glucose reabsorption due to loss of activity of SGLT1 or SGLT2 are in general agreement with the findings in Sglt1/2 knock-out mice. In Sglt2^−/−^ mice the renal glucose reabsorption is reduced to ~50% of that in wild-type mice at euglycemia, and is further reduced with increase in filtered glucose load (Vallon et al., 2011). The knock-out of Sglt1^−/−^ in mice causes a 2–3% decrease in total renal glucose reabsorption (Gorboulev et al., 2012; Powell et al., 2013). The numerical discrepancy in the maximum influence of SGLT1 loss (2–3% in mice vs. 10% in humans) is yet to be understood. It may reflect a real inter-species difference in the contribution of SGLT1, a result secondary to inter-species differences in other physiological factors, or an inter-study variation as well as random errors. Extension of our systems pharmacology model to mice with appropriate physiological parameters could shed light on this issue.

### Explanation to the puzzling moderate inhibition of renal glucose reabsorption by potent SGLT2 inhibitors

Based on the modeling and simulations, it is likely that the apparently moderate inhibition of renal glucose reabsorption induced by potent SGLT2 inhibitors is a combined result of two physiological determinants:

1. *SGLT1 compensation*: Based on the localization and physiological characteristics of SGLT1 and SGLT2 in the kidney, it has been suspected that, with the buildup of glucose along the proximal tubules, SGLT1 will operate more intensely, and hence offset to certain degree the effect of SGLT2 inhibition (Haddish-Berhane et al., 2010; Maurer et al., 2011; Abdul-Ghani et al., 2013). This concept is supported by the modeling and simulation results discussed above. After dapagliflozin treatment in humans, SGLT1 operates at its near maximum capacity and becomes the predominant pathway for glucose reabsorption. Nevertheless, the theoretical maximum inhibition of renal glucose reabsorption is 50–70% in the healthy and T2DM subjects, higher than the observed 30–50%. This disagreement suggests that, besides SGLT1 compensation, there should be additional explanation(s).
2. *Residual activity of SGLT2*: The modeling identified the residual SGLT2 activity to be an additional explanation. Although SGLT2 inhibitors, such as dapagliflozin, canagliflozin, at clinical doses do occupy the majority of SGLT2 and severely suppress SGLT2 activity, they do not completely eliminate SGLT2 activity. This is readily deduced from Equation 2: when the luminal glucose level in PCT rises to several fold higher than K_m_2 resulting from SGLT2 inhibition, the inhibitor exposure has to be several hundred fold of K_i_2, beyond the clinically feasible range, in order to drive the SGLT2-mediated reabsorption rate to near zero. After a treatment with dapagliflozin at 10 mg, there remains at least ~8–10% of residual SGLT2 activity, i.e., 7–11 mmol/h of reabsorption rate, in the healthy and diabetics. This residual activity is still sizeable compared with SGLT1 capacity of 20 mmol/h.

Rieg et al. (2014) recently observed a 56% of lowering of renal glucose reabsorption in mice with complete SGLT2 inhibition, and an entire demolition of reabsorption in mice lacking both SGLT1 and SGLT2. This result confirms the hypothesis of SGLT1 compensation. The extrapolation of this finding to the clinic, however, is complicated by the differences in the experimental conditions in the Rieg et al. (2014) and clinical trials (Komoroski et al., 2009a,b; Devineni et al., 2013; Heise et al., 2013) (see Table S1). While a complete blockage of SGLT2 is likely in the Rieg et al. (2014) with drastically elevated concentration (free plasma concentration at least 10–15-fold higher than *in vitro* IC50) of empagliflozin over the duration of 30 min for UGE collection, in the clinical trials with once daily dosing, it is unlikely to maintain a 100% blockage of SGLT2 throughout a day over which luminal drug concentrations decline and 24 h UGE is collected. Thus, to explain the apparently moderate inhibition of renal glucose reabsorption by potent SGLT2 inhibitors in the clinic, the residual activity of SGLT2 should not be overlooked.

It is worth pointing out that dapagliflozin does severely suppress SGLT2 activity at its approved dose of 10 mg/day, as demonstrated by the simulations (Figure S2) at steady state in a hypothetical healthy subject with a constant plasma glucose level of 100 mg/dL treated with dapagliflozin at various doses. The SGLT2 activity decreases with increase in dose; from 20 mg onward, there is mild further decrease in SGLT2 activity. For SGLT1, its activity is nearly saturated at 10 mg. These results seem to be consistent with previous clinical observations that the UGE effect of dapagliflozin saturates at 20 mg (Komoroski et al., 2009a).

### Effect of an SGLT1/2 dual inhibitor on glucosuria in comparison with a selective SGLT2 inhibitor

It has been a question whether or not an SGLT1/2 dual inhibitor would induce greater glucosuria than a highly selective SGLT2 inhibitor (Chao and Henry, 2010; Abdul-Ghani et al., 2013). Abdul-Ghani et al. (2013) hypothesized that glucosuria induced by an SGLT2 inhibitor with a moderate selectivity over SGLT1 (e.g., capable of inhibiting SGLT1 activity by 30%) may be substantially greater than with a highly selective SGLT2 inhibitor. Using our model, we examined the sensitivity of UGE to V_max_1 and K_i_1 in humans. We found that UGE was mildly to moderately sensitive to V_max_1 but not K_i_1 in the presumably clinically tolerable ranges. The insensitivity to K_i_1 is implied by Equation (2). With a treatment of 10 mg dapagliflozin, the glucose concentration in the PST rises to at least 20-fold of K_m_1. In order to moderately suppress SGLT1-mediated reabsorption through competitive inhibition, the luminal inhibitor exposure has to reach tens of fold of K_i_1, a level that cannot be safely achieved in humans.

Therefore, without the consideration of its effect on intestinal SGLT1, whether or not a dual inhibitor will induce stronger glucosuria than a selective SGLT2 inhibitor is dependent on the mode of interaction between the dual inhibitor and SGLT1. A competitive inhibition of SGLT1 is unlikely to afford the dual inhibitor augmented effect on glucosuria. Other modes of inhibitions (non-competitive or uncompetitive) that attenuate V_max_1 may augment glucosuria mildly to moderately with a dual inhibitor.

In summary, to clarify mechanistically and quantitatively the operating characteristics of SGLT1 and SGLT2 in renal glucose reabsorption, we developed a systems pharmacology model with emphasis on renal glucose filtration, reabsorption, and transfer along the proximal tubules with or without SGLT1/2 inhibition. The model was calibrated using DeFronzo et al. (2013) and evaluated against three other data sets (Mogensen, 1971; Wolf et al., 2009; Polidori et al., 2013). Simulations using this model provided insights into the operating characteristics of SGLTs under normo- and hyperglycemic conditions in the healthy and diabetic state with or without SGLT2 inhibition. The simulations solidified the current concept of the relative contributions of SGLT1/2 to renal glucose reabsorption without the presence of SGLT2 inhibition. Moreover, the simulations elucidated quantitatively the operating characteristics of SGLTs when SGLT2 is inhibited. Further simulations clarified the relationships between SGLT1/2 capacity and renal glucose reabsorption in humans. Based on our modeling and simulations, we propose that the apparent moderate inhibition of renal glucose reabsorption observed clinically with SGLT2 inhibitors is a combined result of two physiological determinants, SGLT1 compensation and residual SGLT2 activity. This model will be valuable in mapping SGLT2 genotype to its functionality, and in predicting, through the incorporation of a plasma glucose-insulin model, the efficacy of an SGLT2 inhibitor in patients with diabetes, especially pediatric patients and patients with type 1 diabetes, for whom clinical data remain scarce.

## Author contributions

Yasong Lu: designed and executed the study, wrote and finalized the report; Steven C. Griffen: designed the study, critically reviewed and approved the report; David W. Boulton: designed the study, critically reviewed and approved the report; Tarek A. Leil: designed the study, reviewed the execution, critically reviewed and approved the report.

### Conflict of interest statement

This study was sponsored by Bristol-Myers Squibb and AstraZeneca. All authors were employees of Bristol-Myers Squibb when the study was being conducted.

## References

[B1] Abdul-Ghani M. A. DeFronzo R. A. Norton L. (2013). Novel hypothesis to explain why SGLT2 inhibitors inhibit only 30–50% of filtered glucose load in humans. Diabetes 62, 3324–3328. 10.2337/db13-0604 24065789 PMC3781482

[B2] Bank N. Aynedjian H. S. (1990). Progressive increases in luminal glucose stimulate proximal sodium absorption in normal and diabetic rats. J. Clin. Invest. 86, 309–316. 10.1172/JCI114700 2365820 PMC296722

[B3] Brown B. P. Hawley H. B. Hessen M. T. (2011). Magill's Medical Guide, Vol. 3: *Fluids and Electrolytes—Kidneys* Pasadena, CA: Salem Press, Incorporated.

[B4] Calado J. Sznajer Y. Metzger D. Rita A. Hogan M. C. Kattamis A.. (2008). Twenty-one additional cases of familial renal glucosuria: absence of genetic heterogeneity, high prevalence of private mutations and further evidence of volume depletion. Nephrol. Dial. Transplant. 23, 3874–3879. 10.1093/ndt/gfn386 18622023

[B5] Chao E. C. Henry R. R. (2010). SGLT2 inhibition—a novel strategy for diabetes treatment. Nat. Rev. Drug. Discov. 9, 551–559. 10.1038/nrd3180 20508640

[B6] DeFronzo R. A. Davidson J. A. Del Prato S. (2012). The role of the kidneys in glucose homeostasis: a new path towards normalizing glycaemia. Diabetes Obes. Metab. 14, 5–14. 10.1111/j.1463-1326.2011.01511.x 21955459

[B7] DeFronzo R. A. Hompesch M. Kasichayanula S. Liu X. Hong Y. Pfister M.. (2013). Characterization of renal glucose reabsorption in response to dapagliflozin in healthy subjects and subjects with type 2 diabetes. Diabetes Care 36, 3169–3176. 10.2337/dc13-0387 23735727 PMC3781504

[B8] Devineni D. Curtin C. R. Polidori D. Gutierrez M. J. Murphy J. Rusch S.. (2013). Pharmacokinetics and pharmacodynamics of canagliflozin, a sodium glucose co-transporter 2 inhibitor, in subjects with type 2 diabetes mellitus. J. Clin. Pharmacol. 53, 601–610. 10.1002/jcph.88 23670707

[B9] Diez-Sampedro A. Wright E. M. Hirayama B. A. (2001). Residue 457 controls sugar binding and transport in the Na^(+)^/glucose cotransporter. J. Biol. Chem. 276, 49188–49194. 10.1074/jbc.M108286200 11602601

[B10] Freckmann G. Hagenlocher S. Baumstark A. Jendrike N. Gillen R. C. Rossner K.. (2007). Continuous glucose profiles in healthy subjects under everyday life conditions and after different meals. J. Diabetes Sci. Technol. 1, 695–703. 10.1177/193229680700100513 19885137 PMC2769652

[B11] Freitas H. S. Anhe G. F. Melo K. F. Okamoto M. M. Oliveira-Souza M. Bordin S.. (2008). Na^(+)^ -glucose transporter-2 messenger ribonucleic acid expression in kidney of diabetic rats correlates with glycemic levels: involvement of hepatocyte nuclear factor-1alpha expression and activity. Endocrinology 149, 717–724. 10.1210/en.2007-1088 17962340

[B12] Gorboulev V. Schurmann A. Vallon V. Kipp H. Jaschke A. Klessen D.. (2012). Na^(+)^-D-glucose cotransporter SGLT1 is pivotal for intestinal glucose absorption and glucose-dependent incretin secretion. Diabetes 61, 187–196. 10.2337/db11-1029 22124465 PMC3237647

[B13] Grempler R. Thomas L. Eckhardt M. Himmelsbach F. Sauer A. Sharp D. E.. (2012). Empagliflozin, a novel selective sodium glucose cotransporter-2 (SGLT-2) inhibitor: characterisation and comparison with other SGLT-2 inhibitors. Diabetes Obes. Metab. 14, 83–90. 10.1111/j.1463-1326.2011.01517.x 21985634

[B14] Haddish-Berhane N. Nucci G. Sawant A. Maurer T. S. Ghosh A. (2010). “A minimal systems pharmacology model of SGLT2/SGLT1 glucose uptake and transport with applications to SGLT2 inhibitors, in Poster Presentation at the 6th International Symposium on Measurement and Kinetics of In Vivo Drug Effects (Noordwijkerhout).

[B15] Heise T. Seewaldt-Becker E. Macha S. Hantel S. Pinnetti S. Seman L.. (2013). Safety, tolerability, pharmacokinetics and pharmacodynamics following 4 weeks' treatment with empagliflozin once daily in patients with type 2 diabetes. Diabetes Obes. Metab. 15, 613–621. 10.1111/dom.12073 23356556

[B16] Hummel C. S. Lu C. Loo D. D. Hirayama B. A. Voss A. A. Wright E. M. (2011). Glucose transport by human renal Na^+^/D-glucose cotransporters SGLT1 and SGLT2. Am. J. Physiol. Cell Physiol. 300, C14–C21. 10.1152/ajpcell.00388.2010 20980548 PMC3023189

[B17] Kleta R. Stuart C. Gill F. A. Gahl W. A. (2004). Renal glucosuria due to SGLT2 mutations. Mol. Genet. Metab. 82, 56–58. 10.1016/j.ymgme.2004.01.018 15110322

[B18] Koeppen B. M. Stanton B. A. (2013). “Chapter 4—renal transport mechanisms: NaCl and water reabsorption along the nephron, in Renal Physiology, 5th Edn., eds Koeppen B. M. Stanton B. A. (Philadelphia, PA: Mosby), 45–71.

[B19] Komoroski B. Vachharajani N. Boulton D. Kornhauser D. Geraldes M. Li L.. (2009a). Dapagliflozin, a novel SGLT2 inhibitor, induces dose-dependent glucosuria in healthy subjects. Clin. Pharmacol. Ther. 85, 520–526. 10.1038/clpt.2008.251 19129748

[B20] Komoroski B. Vachharajani N. Feng Y. Li L. Kornhauser D. Pfister M. (2009b). Dapagliflozin, a novel, selective SGLT2 inhibitor, improved glycemic control over 2 weeks in patients with type 2 diabetes mellitus. Clin. Pharmacol. Ther. 85, 513–519. 10.1038/clpt.2008.250 19129749

[B21] Lam J. T. Martin M. G. Turk E. Hirayama B. A. Bosshard N. U. Steinmann B.. (1999). Missense mutations in SGLT1 cause glucose-galactose malabsorption by trafficking defects. Biochim. Biophys. Acta 1453, 297–303. 10.1016/S0925-4439(98)00109-4 10036327

[B22] Liu J. J. Lee T. DeFronzo R. A. (2012). Why Do SGLT2 inhibitors inhibit only 30–50% of renal glucose reabsorption in humans? Diabetes 61, 2199–2204. 10.2337/db12-0052 22923645 PMC3425428

[B23] Lu Y. Griffen S. C. Boulton D. Lacreta F. Leil T. (2014). “A systems pharmacology model of renal glucose physiology to evaluate the effects of SGLT1 and SGLT2 inhibition in T1DM subjects, in American Conference on Pharmacometrics (Las Vegas, NV).

[B24] Martin M. G. Turk E. Lostao M. P. Kerner C. Wright E. M. (1996). Defects in Na^+^/glucose cotransporter (SGLT1) trafficking and function cause glucose-galactose malabsorption. Nat. Genet. 12, 216–220. 10.1038/ng0296-216 8563765

[B25] Mather A. Pollock C. (2011). Glucose handling by the kidney. Kidney Int. Suppl. 79(Suppl. 120), S1–S6. 10.1038/ki.2010.509 21358696

[B26] Maurer T. S. Ghosh A. Haddish-Berhane N. Sawant-Basak A. Boustany-Kari C. M. She L.. (2011). Pharmacodynamic model of sodium-glucose transporter 2 (SGLT2) inhibition: implications for quantitative translational pharmacology. AAPS J. 13, 576–584. 10.1208/s12248-011-9297-2 21870203 PMC3231856

[B27] Mogensen C. E. (1971). Maximum tubular reabsorption capacity for glucose and renal hemodynamcis during rapid hypertonic glucose infusion in normal and diabetic subjects. Scand. J. Clin. Lab. Invest. 28, 101–109. 10.3109/00365517109090668 5093515

[B28] Moller J. C. Skriver E. (1985). Quantitative ultrastructure of human proximal tubules and cortical interstitium in chronic renal disease (hydronephrosis). Virchows Arch. A Pathol. Anat. Histopathol. 406, 389–406. 10.1007/BF00710231 3925616

[B29] Pfister M. Whaley J. M. Zhang L. List J. F. (2011). Inhibition of SGLT2: a novel strategy for treatment of type 2 diabetes mellitus. Clin. Pharmacol. Ther. 89, 621–625. 10.1038/clpt.2011.16 21346749

[B30] Polidori D. Sha S. Ghosh A. Plum-Morschel L. Heise T. Rothenberg P. (2013). Validation of a novel method for determining the renal threshold for glucose excretion in untreated and canagliflozin-treated subjects with type 2 diabetes mellitus. J. Clin. Endocrinol. Metab. 98, E867–E871. 10.1210/jc.2012-4205 23585665 PMC3706739

[B31] Powell D. R. Dacosta C. M. Gay J. Ding Z. M. Smith M. Greer J.. (2013). Improved glycemic control in mice lacking Sglt1 and Sglt2. Am. J. Physiol. Endocrinol. Metab. 304, E117–E130. 10.1152/ajpendo.00439.2012 23149623

[B32] Rahmoune H. Thompson P. W. Ward J. M. Smith C. D. Hong G. Brown J. (2005). Glucose transporters in human renal proximal tubular cells isolated from the urine of patients with non-insulin-dependent diabetes. Diabetes 54, 3427–3434. 10.2337/diabetes.54.12.3427 16306358

[B33] Rieg T. Masuda T. Gerasimova M. Mayoux E. Platt K. Powell D. R.. (2014). Increase in SGLT1-mediated transport explains renal glucose reabsorption during genetic and pharmacological SGLT2 inhibition in euglycemia. Am. J. Physiol. Renal. Physiol. 306, F188–F193. 10.1152/ajprenal.00518.2013 24226519 PMC3920019

[B34] Santer R. Kinner M. Lassen C. L. Schneppenheim R. Eggert P. Bald M.. (2003). Molecular analysis of the SGLT2 gene in patients with renal glucosuria. J. Am. Soc. Nephrol. 14, 2873–2882. 10.1097/01.ASN.0000092790.89332.D2 14569097

[B35] Tabatabai N. M. Sharma M. Blumenthal S. S. Petering D. H. (2009). Enhanced expressions of sodium-glucose cotransporters in the kidneys of diabetic Zucker rats. Diabetes Res. Clin. Pract. 83, e27–e30. 10.1016/j.diabres.2008.11.003 19095325 PMC2652566

[B36] Thelwall P. E. Taylor R. Marshall S. M. (2011). Non-invasive investigation of kidney disease in type 1 diabetes by magnetic resonance imaging. Diabetologia 54, 2421–2429. 10.1007/s00125-011-2163-z 21533898

[B37] Vallon V. (2011). Molecular determinants of renal glucose reabsorption. Focus on “Glucose transport by human renal Na^+^/D-glucose cotransporters SGLT1 and SGLT2.” Am. J. Physiol. Cell Physiol. 300, C6–C8. 10.1152/ajpcell.00444.2010 21048164 PMC3023181

[B38] Vallon V. Gerasimova M. Rose M. A. Masuda T. Satriano J. Mayoux E.. (2014). SGLT2 inhibitor empagliflozin reduces renal growth and albuminuria in proportion to hyperglycemia and prevents glomerular hyperfiltration in diabetic Akita mice. Am. J. Physiol. Renal. Physiol. 306, F194–F204. 10.1152/ajprenal.00520.2013 24226524 PMC3920018

[B39] Vallon V. Platt K. A. Cunard R. Schroth J. Whaley J. Thomson S. C.. (2011). SGLT2 mediates glucose reabsorption in the early proximal tubule. J. Am. Soc. Nephrol. 22, 104–112. 10.1681/ASN.2010030246 20616166 PMC3014039

[B40] Vallon V. Thomson S. C. (2012). Renal function in diabetic disease models: the tubular system in the pathophysiology of the diabetic kidney. Annu. Rev. Physiol. 74, 351–375. 10.1146/annurev-physiol-020911-153333 22335797 PMC3807782

[B41] Washburn W. N. Poucher S. M. (2013). Differentiating sodium-glucose co-transporter-2 inhibitors in development for the treatment of type 2 diabetes mellitus. Expert. Opin. Investig. Drugs 22, 463–486. 10.1517/13543784.2013.774372 23452053

[B42] Wolf S. Rave K. Heinemann L. Roggen K. (2009). Renal glucose excretion and tubular reabsorption rate related to blood glucose in subjects with type 2 diabetes with a critical reappraisal of the “renal glucose threshold” model. Horm. Metab. Res. 41, 600–604. 10.1055/s-0029-1220723 19418417

[B43] Wright E. M. (2001). Renal Na^(+)^-glucose cotransporters. Am. J. Physiol. Renal. Physiol. 280, F10–F18. Available online at: http://ajprenal.physiology.org/content/280/1/F10 11133510 10.1152/ajprenal.2001.280.1.F10

[B44] Yamaguchi K. Kato M. Ozawa K. Kawai T. Yata T. Aso Y.. (2012). Pharmacokinetic and pharmacodynamic modeling for the effect of sodium-glucose cotransporter inhibitors on blood glucose level and renal glucose excretion in db/db mice. J. Pharm. Sci. 101, 4347–4356. 10.1002/jps.23302 22927193

[B45] Yamaguchi K. Kato M. Suzuki M. Asanuma K. Aso Y. Ikeda S.. (2011). Pharmacokinetic and pharmacodynamic modeling of the effect of an sodium-glucose cotransporter inhibitor, phlorizin, on renal glucose transport in rats. Drug Metab. Dispos. 39, 1801–1807. 10.1124/dmd.111.040048 21712434

[B46] Yu L. Lv J. C. Zhou X. J. Zhu L. Hou P. Zhang H. (2011). Abnormal expression and dysfunction of novel SGLT2 mutations identified in familial renal glucosuria patients. Hum. Genet. 129, 335–344. 10.1007/s00439-010-0927-z 21165652

[B47] Zambrowicz B. Ding Z. M. Ogbaa I. Frazier K. Banks P. Turnage A.. (2013). Effects of LX4211, a dual SGLT1/SGLT2 inhibitor, plus sitagliptin on postprandial active GLP-1 and glycemic control in type 2 diabetes. Clin. Ther. 35, 273–285. 10.1016/j.clinthera.2013.01.010 23433601

