# Supplementary material for: Use of systems pharmacology modeling to elucidate the operating characteristics of SGLT1 and SGLT2 in renal glucose reabsorption in humans
Source: Front Pharmacol. 2014 Dec 10;5:274. doi: 10.3389/fphar.2014.00274 (PMC4261707; doi:10.3389/fphar.2014.00274)
Supplement: Supplementary file 1 [file Presentation1.PDF]

Supplementary Materials

Use systems pharmacology modeling to elucidate the operating characteristics of SGLT1 and SGLT2 in renal glucose reabsorption in humans

Yasong Lu, Steven C. Griffen, David W. Boulton, Tarek Leil

Key equations of the model

(1) Rate of luminal glucose reabsorption (R) mediated by SGLT2 in sub-segments of proximal convoluted tubules (PCT1-6) at baseline

$$R_j = \frac{\left(V_{\max 2}/6\right) \times C_{glu,j}}{K_{m2} + C_{glu,j}} \qquad \text{(Equation S1)}$$

where j is an index for sub-segments PCT1-6, Vmax2 is the total capacity of SGLT2, which is uniformly distributed to PCT1-6, Km2 is the affinity of glucose to SGLT2, and Cglu is luminal glucose concentration in a PCT sub-segment.

(2) Rate of luminal glucose reabsorption (R) mediated by SGLT1 in sub-segments of proximal straight tubules (PST1-3) at baseline

$$R_j = \frac{\left(V_{\max 1}/3\right) \times C_{glu,j}}{K_{m1} + C_{glu,j}} \qquad \text{(Equation S2)}$$

where j is an index for sub-segments PST1-3, Vmax1 is the total capacity of SGLT1, which is uniformly distributed to PST1-3, Km1 is the affinity of glucose to SGLT1, and Cglu is luminal glucose concentration in a PST sub-segment.

(3) Rate of luminal glucose reabsorption (R*) mediated by SGLT2 in PCT1-6 after dapagliflozin treatment

$$R_j^* = \frac{\left(V_{\max 2}/6\right) \times C_{glu,j}}{K_{m2} \times \left(1 + \frac{C_{drug,j}}{K_{i2}}\right) + C_{glu,j}} \qquad \text{(Equation S3)}$$

where Ki2 is the affinity of dapagliflozin for SGLT2. The competitive inhibitory effect of dapagliflozin on the reabsorption rate is reflected by the multiplication factor (1+Cdrug,j/Ki2). Vmax2 is not affected by dapagliflozin.

(4) Rate of luminal glucose reabsorption (R*) mediated by SGLT1 in PST1-3 after dapagliflozin treatment

$$R_j^* = \frac{\left(V_{\max 1}/3\right) \times C_{glu,j}}{K_{m1} \times \left(1 + \frac{C_{drug,j}}{K_{i1}}\right) + C_{glu,j}} \qquad \text{(Equation S4)}$$

where Ki1 is the affinity of dapagliflozin for SGLT1. The competitive inhibitory effect of dapagliflozin, albeit minor, on the reabsorption rate is reflected by the multiplication factor (1+Cdrug,j/Ki1). Vmax1 is not affected by dapagliflozin.

Table S1. Comparison of study conditions in the Rieg et al. (2014) in mice and the clinical trials (Komoroski et al., 2009a;Komoroski et al., 2009b;Devineni et al., 2013;Heise et al., 2013) demonstrating pharmacodynamic effects of SGLT2 inhibitors

Study conditions	Rieg et al.	Clinical trials
Dosing regimen	Drug in diet, or drug in diet + an additional intraperitoneal dose	Single oral dose, or once a day oral dose for a week or longer
Duration of UGE collection	30 min	24 h intervals
Timing of UGE collection relative to drug concentration profile	At peak concentration, when drug concentration/Ki2 ratio is high	From peak to trough, where drug concentration/Ki2 ratio drops from the maximum to the minimum
Possibility of reaching 100% inhibition of SGLT2 within the duration of UGE collection	High	Low

Figure S1. Glucose concentrations in tubular subsegments (PCT1-6 and PST1-3) in T2DM patients at baseline and treated with dapagliflozin under the experimental conditions in DeFronzo et al. (2013).

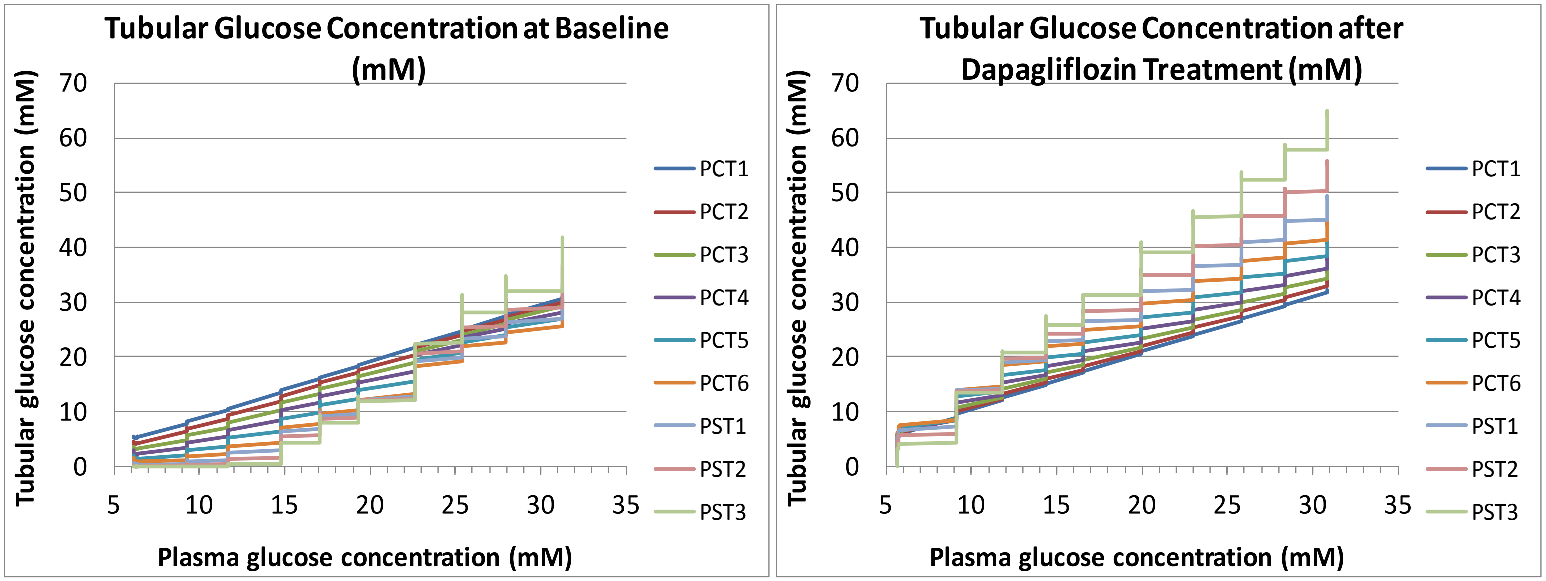

Note: PCT1-6: sub-segments 1-6 of proximal convoluted tubules; PST1-3: sub-segments 1-3 of proximal straight tubules

Figure S2. Model-calculated steady-state rate of renal glucose reabsorption mediated by SGLT1 or SGLT2 at various dapagliflozin doses in a hypothetical healthy subject with a constant plasma glucose level of 100 mg/dL.

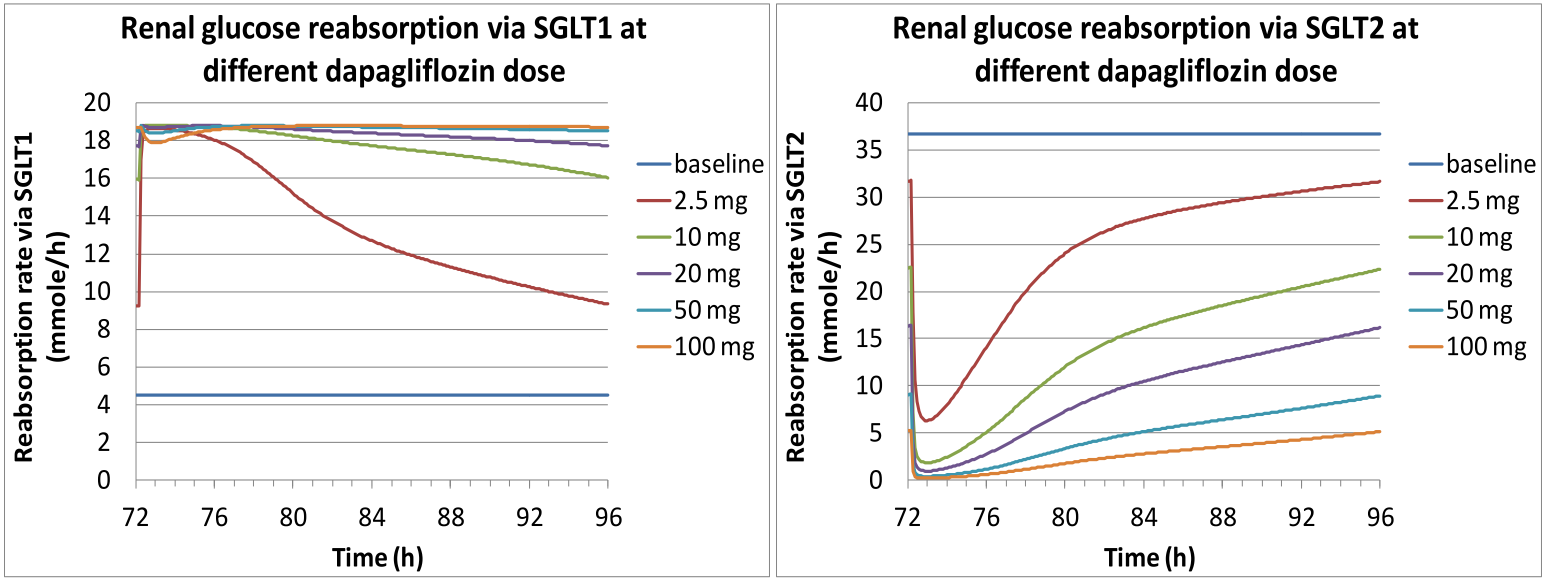

References:

Defronzo, R.A., Hompesch, M., Kasichayanula, S., Liu, X., Hong, Y., Pfister, M., Morrow, L.A., Leslie, B.R., Boulton, D.W., Ching, A., Lacreta, F.P., and Griffen, S.C. (2013). Characterization of Renal Glucose Reabsorption in Response to Dapagliflozin in Healthy Subjects and Subjects With Type 2 Diabetes. *Diabetes Care* 36**,** 3169-3176.

Devineni, D., Curtin, C.R., Polidori, D., Gutierrez, M.J., Murphy, J., Rusch, S., and Rothenberg, P.L. (2013). Pharmacokinetics and pharmacodynamics of canagliflozin, a sodium glucose co-transporter 2 inhibitor, in subjects with type 2 diabetes mellitus. *J Clin Pharmacol* 53**,** 601-610.

Heise, T., Seewaldt-Becker, E., Macha, S., Hantel, S., Pinnetti, S., Seman, L., and Woerle, H.J. (2013). Safety, tolerability, pharmacokinetics and pharmacodynamics following 4 weeks' treatment with empagliflozin once daily in patients with type 2 diabetes. *Diabetes Obes Metab* 15**,** 613-621.

Komoroski, B., Vachharajani, N., Boulton, D., Kornhauser, D., Geraldes, M., Li, L., and Pfister, M. (2009a). Dapagliflozin, a novel SGLT2 inhibitor, induces dose-dependent glucosuria in healthy subjects. *Clin Pharmacol Ther* 85**,** 520-526.

Komoroski, B., Vachharajani, N., Feng, Y., Li, L., Kornhauser, D., and Pfister, M. (2009b). Dapagliflozin, a novel, selective SGLT2 inhibitor, improved glycemic control over 2 weeks in patients with type 2 diabetes mellitus. *Clin Pharmacol Ther* 85**,** 513-519.

Rieg, T., Masuda, T., Gerasimova, M., Mayoux, E., Platt, K., Powell, D.R., Thomson, S.C., Koepsell, H., and Vallon, V. (2014). Increase in SGLT1-mediated transport explains renal glucose reabsorption during genetic and pharmacological SGLT2 inhibition in euglycemia. *Am J Physiol Renal Physiol* 306**,** F188-193.
